# Peroxiredoxin 4: a multifunctional biomarker worthy of further exploration

**DOI:** 10.1186/1741-7015-9-137

**Published:** 2011-12-23

**Authors:** Janin Schulte

**Affiliations:** 1 Department of Research and Development, BRAHMS GmbH, Part of Thermo Fisher Scientific, 16761 Hennigsdorf, Germany

## Abstract

Currently, there is much interest in identifying clinically relevant biomarkers, as they have the potential to be high utility non-invasive tools for early diagnosis and reliable patient monitoring in numerous conditions. Since its discovery almost 15 years ago, research on the ubiquitous antioxidant enzyme peroxiredoxin 4 (Prx4) has culminated in the recognition that Prx4 levels are different in blood drawn from the healthy general population and patients with acute or chronic diseases. In this commentary, the most striking research data from different *in vitro *approaches, animal models and human observational studies are discussed collectively, highlighting the clinical importance of Prx4 as a multifunctional staging and prognosis biomarker. In this context, the oxidative state of patients may be reflected by intra- and extracellular Prx4 levels, redox state, oligomerization and nitro-oxidative modifications of the enzyme. A consolidated model of the potential role and origin of circulating Prx4 is presented to stimulate further investigations in light of the current biomarker situation.

## Background

Physiologically important processes such as redox signaling and host defense depend on balanced amounts of reactive oxygen species (ROS), including free radicals and stable oxidizing molecules, for example, hydrogen peroxide (H_2_O_2_). In conditions of oxidative stress, increased ROS levels overwhelm antioxidant protection mechanisms, resulting in the development and progression of various diseases [1].

Peroxiredoxin 4 (Prx4; synonyms: AOE372, TRANK) is a ubiquitously expressed member of the peroxiredoxin family that is localized in the endoplasmic reticulum (ER) and extracellular space [2,3], with highest expression in the pancreas, liver and heart, and lowest expression in blood leukocytes and the brain [2,4]. The enzyme diminishes oxidative stress by reducing hydrogen peroxide to water in a thiol-dependent catalytic cycle [5] and has been linked to the regulation of the key pro-inflammatory transcription factor, nuclear factor kappa B (NF-κB) [2,4,6]. In addition, redox-dependent and reversible conversion of homologous Prx1-4 from disulfide-linked homodimers to higher-order multimers and back provides a versatile mechanism to switch between peroxidase and chaperone activity, enabling interaction with binding partners including stress-responsive kinases, membrane proteins and immune-modulators, fine-tuning of H_2_O_2 _signaling [7] and, as published most recently, circadian clock oscillations [8]. Disturbances in the aforementioned fundamental processes clearly result in numerous pathological conditions that could potentially be reflected by the measurement of involved Prx.

Among the six Prx isoforms in mammals, Prx4 emerges as the most attractive, easily accessible biomarker candidate as it was initially designated as a secretable peroxidase [2,9], has recently been identified in the circulation of healthy and diseased individuals [10,11] and, finally, has been linked to morbidity and mortality in patients with sepsis in the intensive care unit and patients with non-specific complaints in the emergency department [12,13]. In addition, several studies have shown that the expression or oxidation state of Prx4 changes under pathological conditions such as cancer [14-20], diabetes [21-23] and infection [4,6,24-27].

Fast evolving genomic and proteomic studies constantly provide many new biomarker candidates, unavoidably lacking a differentiated evaluation of single candidates and possibly misdirecting promising fields of application. Hence, this commentary intends to discuss the available data on Prx4 at an early stage, focusing on the relevant clinical evidence that indicates strong biomarker potential in a number of conditions in critical care and the emergency room.

## Discussion

### Clinical significance of Prx4 detection

Prx4 should be of special interest to clinicians as it may represent a new marker candidate that could be highly valuable in predicting the prognosis of an adverse outcome to risk stratify patients and to monitor therapy. Indeed, serum and expression levels, as well as post-translational modifications, of the enzyme have been linked to a number of different diseases, ranging from primary care to advanced critical care topics (see Additional file 1). The biomarker performance of Prx4 in these patients is truly encouraging, given the high heterogeneity and potential confounders, such as co-morbidities and medication. Indeed, Prx4 expression in the mouse liver was responsive to different drugs, including the analgesic paracetamol and the chemotherapeutic etoposide phosphate [28]. Altogether, Prx4 is assumed to be important in many single settings, which is very promising, but requires further validation. The results found by Chang and colleagues [10], for instance, are methodologically questionable because they are based on an immunoassay that worked with diluted patient plasma directly coated to the solid phase, which strongly limits specific antibody binding. Moreover, findings from different cancer studies are in part contradictory, concluding either good [16,18] or bad prognosis [14,19] from increased Prx4 expression in tumor tissue. Specifically, sepsis [29], cancer [30] and cardiovascular disease [31] are candidate diseases where Prx4 measurement may be of particular use as they share redox and inflammatory dysregulation and frequently lack valid severity and risk assessment by non-invasive biomarkers.

### Perspectives for Prx4 testing methods

An improved understanding of the redox regulation, structural assembly and activity of Prx4 would provide a better insight into analyzing Prx4 as a biomarker in tissue or serum. Conoidin A, a specific Prx activity inhibitor, is currently under evaluation [32]. Tavender and Bulleid found Prx4 oxidation to be indicative of the degree of oxidative stress in the ER [3]. Atherosclerotic plaque development (Full *et al*., unpublished data), viral infection of A549 lung adenocarcinoma epithelial cells [25] and stimulation of human umbilical vein endothelial cells (HUVEC) with H_2_O_2 _and hydroperoxides [33] resulted in oxidation and a shifted isoelectric point of Prx4, respectively. Moreover, selective changes for either a putative 31-kDa precursor or 27-kDa secretable Prx4 have been reported in lung cancer [34] and spermatogenesis disorders [35,36]. Consequently, detection of Prx4 hyperoxidation and oligomerization [3,25,37,38] could be a meaningful, albeit challenging, measure of thiol-redox imbalances and should be integrated into Prx4 analysis. Existing expression data, however, require careful interpretation in light of a newly discovered 29.5-kDa splice variant of Prx4 [39] that cannot be recognized by the antibodies used in the immunoassay that has been developed for the detection of Prx4 dimers and oligomers in serum [11].

### Origin and drivers of circulating Prx4

**Figure 1:**
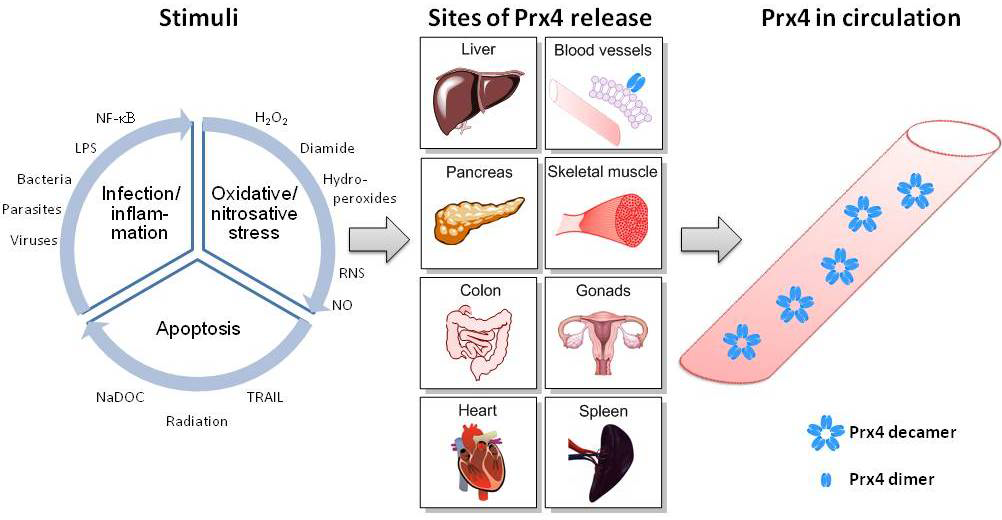
**Potential origin and regulation of peroxiredoxin 4**. Peroxiredoxin 4 (Prx4) is known to switch between dimer and pentadimeric decamer structure [3], with potential decameric structure having been identified in circulation [11]. It has been shown that Prx4 redox-dependently binds to the cell surface of human umbilical vein endothelial cells [9]. Specific and non-specific stimulation of various tissues has influenced Prx4 expression, oxidation or oligomerization and may ultimately result in secretion of Prx4. H_2_O_2_: hydrogen peroxide; LPS: lipopolysaccharide; NaDOC: sodium deoxycholate; NF-κB: nuclear factor-kappa B; NO: nitric oxide; Prx4: peroxiredoxin 4; RNS: reactive nitrogen species; TRAIL: tumor necrosis factor-related apoptosis-inducing ligand.

The amount of secreted Prx4 is most likely proportional to the initial intracellular expression levels. With exceptions [40], it has been shown in different *in vivo *models of infection that Prx4 expression was further potentiated in organs with strong endogenous expression of the enzyme [26,41]. Consequently, tissue with high basal or up-regulated cellular expression of Prx4 likely represents a source for circulating Prx4 (Figure 1). Okado-Matsumoto and colleagues hypothesized that the enzyme is bound to the endothelial cells of blood vessels and released in response to a changed redox environment [9], matched by the high expression of Prx4 in endothelial cells [2]. There is sufficient further evidence that augmented Prx4 serum levels may be a result of secretion in response to pro-oxidant and pro-inflammatory signaling cascades, possibly related to regulation of NF-κB [2,4,6] (Figure 1). Indeed, Prx4 serum levels are correlated with endogenous antioxidants (albumin, bilirubin) and markers of inflammation (C-reactive protein, interleukin-6) in critically ill patients [13]. Moreover, changes in Prx4 expression or oxidation resulted from viral infection [4,6,24-27] and oxidative stimulation [33,38,42,43]*in vitro *and *in vivo*. Preliminary data from Madin-Darby canine kidney strain I cells stably transfected with Prx4 revealed a significant dose-dependent increase of Prx4 secretion after stimulation with H_2_O_2 _(Schulte *et al*., unpublished data). In addition, intracellular Prx4 was associated with nitrotyrosine expression in bladder and ovarian cancer [14,16,44] and nitric oxide-dependent regulation in stimulated macrophages [43], suggesting nitrosative stress as another promoter of Prx4 secretion. Ultimately, circulating Prx4 may originate from non-specific membrane leakage due to tissue damage and apoptotic cell death, representing a consequence of the aforementioned stress stimuli (Figure 1).

### Consequences of Prx4 modification

**Figure 2:**
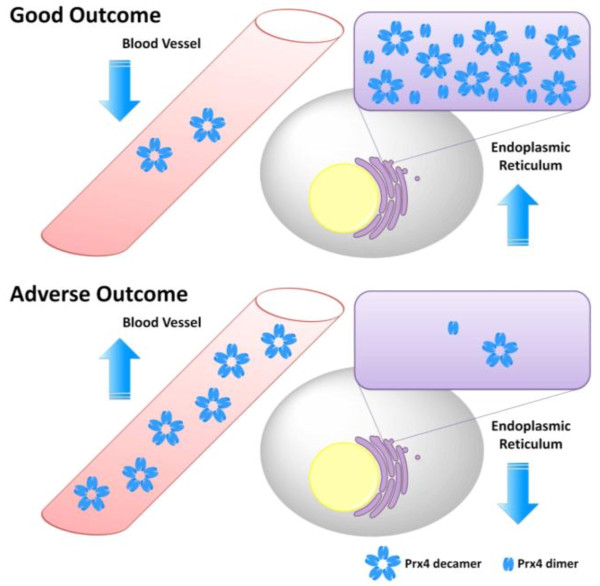
**Speculative model of unfavorable peroxiredoxin 4 translocation**. Intracellular peroxiredoxin 4 (Prx4) is localized in the endoplasmic reticulum where it switches between dimer and pentadimeric decamer structure [3]. A potential decameric structure has been proposed for extracellular Prx4 circulating in the blood [11]. A 'good outcome', that is, an improvement of or a protection against pathological processes, could be achieved by high cellular Prx4 levels. Low endogenous Prx4 serum levels, corresponding to non-secreted high intracellular Prx4 levels, imply a non-serious disease course. Accordingly, decreased intracellular Prx4 levels or elevated endogenous serum levels of the enzyme are indicative of 'adverse outcome', that is, a more severe disease course or a higher mortality rate.

Intracellular Prx4 is mainly localized in the ER, where it contributes to redox and disulfide regulation [3], whereas no definite function has yet been assigned for extracellular Prx4 [6,9]. Preincubation of HUVEC with reduced Prx4 resulted in improved cell viability after H_2_O_2 _treatment [9]. Protective anti-inflammatory effects have been reported for Prx4, when injected or overexpressed during infection [4,6,26]. In addition, Prx4 expression has been shown to be relevant for the antioxidant effects of natural compounds [42,45,46]. Chaperone activity in the extracellular space is conceivable, but requires experimental confirmation [7,47]. A recent publication by Palande and colleagues indirectly linked Prx4 to the innate immune system by showing that ER-localized Prx4 has an inhibitory effect on granulocyte colony-stimulating factor, which is a key regulator of neutrophil production [48]. However, Prx4 secretion could lead to a loss of intracellular or membrane-bound Prx4, which would result in a changed capacity to remove H_2_O_2_, to regulate redox mechanisms or to participate in signaling pathways in the cellular vicinity. The resulting cellular oxidative stress and disturbed signaling would initiate and exacerbate pathological processes, largely increasing the probability of adverse outcome and death. On this basis, a speculative model is suggested, concluding that increased serum levels of Prx4 as a result of unfavorable Prx4 translocation could indicate higher morbidity (Figure 2). Indeed, elevated Prx4 serum levels have been linked to adverse outcome and higher mortality in critical care and emergency settings [12,13] as well as in the general population (Abbasi *et al*., unpublished data) and type 2 diabetes mellitus patients (Alkhalaf *et al*., unpublished data). In addition, elevated intracellular Prx4 levels in carcinoma tissue are associated with improved survival [16,18], in contrast to the fact that tumor cells escape apoptosis by increased Prx4 expression [49-52]. This is in better agreement with results from other settings reporting higher mortality in cancer patients with increased Prx4 expression levels in tumor tissue [14,19]. Finally, there is evidence that Prx isoforms and other antioxidants can substitute for Prx4 [14,18,25,34,53], resulting in compensation for functional consequences of Prx4 release.

## Conclusions

It seems reasonable by now to expand basic clinical research from exclusive expression studies to mechanisms of Prx4 release, oligomerization and oxidative modification in order to identify relevant biochemical interrelations and refine the actual biomarker value of Prx4. Present data indicate that Prx4, either intracellular or extracellular, has the potential to serve as a biomarker for both the early staging of disease severity and the prediction of the future disease course in different pathologies that involve dysregulation of the redox system, for example, as evident during inflammatory processes. Accordingly, it is recommended that promising results from recent studies be validated and clinical trials be performed in new indications to ultimately assess the overall use of Prx4 for risk stratification and therapy monitoring in comparison with existing standards.

## Competing interests

JS is an employee of BRAHMS GmbH, Part of Thermo Fisher Scientific, who have developed and patented the Prx4 immunoassay.

## Pre-publication history

The pre-publication history for this paper can be accessed here:

http://www.biomedcentral.com/1741-7015/9/137/prepub

## Supplementary Material

Additional file 1 **Peroxiredoxin 4 in clinical indications**. This overview does not presume to be complete. Indeed, cited cancer settings are limited to subgroups with important clinical findings beyond changes in peroxiredoxin 4 expression. Unless otherwise mentioned, the term 'Prx4' refers to intracellular protein levels. Click here for file
