# Supplementary material for: Peroxiredoxin 4: a multifunctional biomarker worthy of further exploration
Source: BMC Med. 2011 Dec 23;9:137. doi: 10.1186/1741-7015-9-137 (PMC3260115; doi:10.1186/1741-7015-9-137)
Supplement: Additional file 1 — Peroxiredoxin 4 in clinical indications. This overview does not presume to be complete. Indeed, cited cancer settings are limited to subgroups with important clinical findings beyond changes in peroxiredoxin 4 expression. Unless otherwise mentioned, the term 'Prx4' refers to intracellular protein levels. [file 1741-7015-9-137-S1.DOC]

| **Indication** | **Setting** | **References** |
| --- | --- | --- |
| Autoimmune diseases | RA^a^: increased Prx4 in human plasma and synovial fluid of RA vs. OA and AS patients and healthy controls | [1] |
|  | SLE, RA, Behçet disease: autoantibodies to Prx4 in human serum | [2] |
|  | Celiac disease: increased Prx4 in human duodenal biopsies of Marsh stages II-III vs. 0-I and diagnosis-negative controls | [3] |
| Cancer | Bladder cancer: positive Prx4 in human urothelial bladder carcinomas; association with tumor stage, size and long-term mortality | [4] |
|  | Lung cancer: increased Prx4 in human lung adenocarcinomas vs. normal lung specimens (27 and 31 kDa form); association with tumor differentiation | [5, 6] |
|  | Ovarian cancer: positive Prx4 in human invasive epithelial ovarian carcinomas; association with long-term mortality | [7] |
|  | Breast cancer: increased Prx4 in human breast carcinomas vs. healthy breast specimens; association with tumor differentiation, progesterone receptor status and long-term mortality | [8, 9] |
|  | Oral cavity squamous cell carcinoma: increased Prx4 in tumor vs. normal epithelia; increased Prx4 in metastatic vs. primary lymph node tumors; association with pathologic nodal status and long-term mortality; decreased cell migration and invasiveness by downregulation of Prx4 | [10] |
|  | Prostate cancer: increased Prx4 in human prostate cancer cell lines and prostate carcinomas vs. normal prostate specimens; association with age, tumor stage and Gleason score | [11] |
|  | Colorectal cancer: increased Prx4 transcript in liver metastatic vs. non-metastatic tumors | [12] |
| Cardiovascular diseases | Failing myocardium: decreased Prx4 in human failing vs. non-failing left ventricular tissue | [13] |
|  | Cardiac hypertrophy: decreased Prx4 in hearts of heterozygous SOD2-knockout vs. wildtype mice, increased cardiac Prx4 in knockout animals after physical exercise | [14] |
|  | Carotid disease: increased Prx4 oxidation in human plaque vs. healthy smooth muscle cells | (Full et al. 2010, unpublished data) |
| Cerebrovascular diseases | Middle cerebral artery occlusion: increased Prx4 in ipsilateral white matter tracts of rats after infusion of human umbilical cord blood | [15] |
| Gastrointestinal diseases | Mutation of hemochromatosis gene *HFE* in neuronal cells: increased Prx4 transcript in mutant vs. wildtype cells | [16] |
|  | Drug-induced liver steatosis: decreased Prx4 in rat liver membrane fraction of treated vs. control group | [17] |
|  | Acute pancreatitis: increased serum Prx4 in patients with severe vs. mild AP | (Weiss et al. 2010, unpublished data) |
| Infectious diseases | SIRS, sepsis, severe sepsis and septic shock in the ICU: increased serum Prx4 in patients vs. healthy controls; association with disease severity and 28-day mortality | [18] |
|  | Rabies virus infection in neuroblastoma cells: strain-dependently increased Prx4 following infection | [19] |
|  | *Vibrio* infection in fish: increased Prx4 in spleen of infected vs. control animals; enhanced survival by injected Prx4 | [20] |
|  | Respiratory syncytial virus infection in lung cells: shifted isoelectric point of Prx4 following infection | [21] |
|  | *Schistosoma mansoni* infection in snails: increased Prx4 in hepatopancreas of resistant vs. susceptible animals | [22] |
|  | Hepatitis delta virus infection in hepatic cells: increased Prx4 in virus-transfected vs. control cells | [23] |
|  | HIV infection in T cells: decreased Prx4 in infected vs. control cells; reduced viral replication by overexpression of Prx4 | [24] |
| Metabolic diseases | Diet-induced beta cell dysfunction: increased Prx4 transcript in pancreatic islets of high-fat vs. carbohydrate-free high-fat diet mice | [25] |
|  | Streptozotocin-induced T1DM: decreased Prx4 in pancreas following STZ injection in rodents | [26, 27] |
|  | T2DM: association of increased serum Prx4 with long-term cardiovascular and all-cause mortality in patients | (Alkhalaf et al. 2011, unpublished data) |
| Urogenital diseases | Azoospermia: decreased 31 kDa Prx4 precursor in testis biopsies of azoospermic vs. fertile men | [28] |
|  | Cryptorchidism: decreased 31 kDa Prx4 precursor in testis 14 days after artificial crytorchidism in mice | [29] |
|  | Renal ischemia and reperfusion: increased Prx4 in contralateral kidney of treated vs. control mice | [30] |
| Others | Non-specific complaints in the ED: increased serum Prx4 in patients vs. healthy controls; association with 30-day mortality | [31] |
|  | General population, Netherlands (PREVEND): increased serum Prx4 in metabolic syndrome, cardiovascular disease and T2DM; association with 10-years risk of T2DM incidence, cardiovascular events and death | (Abbasi et al. 2011, unpublished data) |

References

1. Chang X, Cui Y, Zong M, Zhao Y, Yan X, Chen Y, Han J: **Identification of proteins with increased expression in rheumatoid arthritis synovial tissues**. *J Rheumatol* 2009, **36**(5):872-880.

2. Karasawa R, Ozaki S, Nishioka K, Kato T: **Autoantibodies to peroxiredoxin I and IV in patients with systemic autoimmune diseases**. *Microbiol Immunol* 2005, **49**(1):57-65.

3. Simula MP, Cannizzaro R, Canzonieri V, Pavan A, Maiero S, Toffoli G, De Re V: **PPAR signaling pathway and cancer-related proteins are involved in celiac disease-associated tissue damage**. *Mol Med* 2010, **16**(5-6):199-209.

4. Soini Y, Haapasaari KM, Vaarala MH, Turpeenniemi-Hujanen T, Kärjä V, Karihtala P: **8-hydroxydeguanosine and nitrotyrosine are prognostic factors in urinary bladder carcinoma**. *Int J Clin Exp Pathol* 2011, **4**(3):267-275.

5. Chen G, Gharib TG, Huang CC, Thomas DG, Shedden KA, Taylor JM, Kardia SL, Misek DE, Giordano TJ, Iannettoni MD, [Orringer MB](http://www.ncbi.nlm.nih.gov/pubmed?term="Orringer MB"%5BAuthor%5D), [Hanash SM](http://www.ncbi.nlm.nih.gov/pubmed?term="Hanash SM"%5BAuthor%5D), [Beer DG](http://www.ncbi.nlm.nih.gov/pubmed?term="Beer DG"%5BAuthor%5D): **Proteomic analysis of lung adenocarcinoma: identification of a highly expressed set of proteins in tumors**. *Clin Cancer Res* 2002, **8**(7):2298-2305.

6. Lehtonen ST, Svensk AM, Soini Y, Paakko P, Hirvikoski P, Kang SW, Saily M, Kinnula VL: **Peroxiredoxins, a novel protein family in lung cancer**. *International journal of cancer* 2004, **111**(4):514-521.

7. Karihtala P, Soini Y, Vaskivuo L, Bloigu R, Puistola U: **DNA adduct 8-hydroxydeoxyguanosine, a novel putative marker of prognostic significance in ovarian carcinoma**. *Int J Gynecol Cancer* 2009, **19**(6):1047-1051.

8. Karihtala P, Kauppila S, Soini Y, Jukkola-Vuorinen A: **Oxidative stress and counteracting mechanisms in hormone receptor positive, triple-negative and basal-like breast carcinomas**. *BMC Cancer* 2011, **11**(1):262.

9. Karihtala P, Mantyniemi A, Kang SW, Kinnula VL, Soini Y: **Peroxiredoxins in breast carcinoma**. *Clin Cancer Res* 2003, **9**(9):3418-3424.

10. Chang KP, Yu JS, Chien KY, Lee CW, Liang Y, Liao CT, Yen TC, Lee LY, Huang LL, Liu SC, [Chang YS](http://www.ncbi.nlm.nih.gov/pubmed?term="Chang YS"%5BAuthor%5D), [Chi LM](http://www.ncbi.nlm.nih.gov/pubmed?term="Chi LM"%5BAuthor%5D): **Identification of PRDX4 and P4HA2 as metastasis-associated proteins in oral cavity squamous cell carcinoma by comparative tissue proteomics of microdissected specimens using iTRAQ technology**. *J Proteome Res* 2011, Nov 4;10(11):4935-47.

11. Basu A, Banerjee H, Rojas H, Martinez SR, Roy S, Jia Z, Lilly MB, De León M, Casiano CA: **Differential expression of peroxiredoxins in prostate cancer: consistent upregulation of PRDX3 and PRDX4**. *Prostate* 2011, **71**(7):755-765.

12. Li M, Lin YM, Hasegawa S, Shimokawa T, Murata K, Kameyama M, Ishikawa O, Katagiri T, Tsunoda T, Nakamura Y, [Furukawa Y](http://www.ncbi.nlm.nih.gov/pubmed?term="Furukawa Y"%5BAuthor%5D):**Genes associated with liver metastasis of colon cancer, identified by genome-wide cDNA microarray.** *Int J Oncol* 2004, **24**(2):305-312.

13. Brixius K, Schwinger RH, Hoyer F, Napp A, Renner R, Bolck B, Kumin A, Fischer U, Mehlhorn U, Werner S, [Bloch W](http://www.ncbi.nlm.nih.gov/pubmed?term="Bloch W"%5BAuthor%5D):**Isoform-specific downregulation of peroxiredoxin in human failing myocardium**. *Life sciences* 2007, **81**(10):823-831.

14. Richters LK, Lange N, Renner R, Treiber N, Ghanem A, Tiemann K, Scharffetter-Kochanek K, Bloch W, Brixius K: **Exercise-induced adaptations of cardiac Redox homeostasis and remodelling in heterozygous SOD2-knockout mice**. *J Appl Physiol* 2011, **[Epub ahead of print]**.

15. Rowe DD, Leonardo CC, Hall AA, Shahaduzzaman MD, Collier LA, Willing AE, Pennypacker KR: **Cord blood administration induces oligodendrocyte survival through alterations in gene expression**. *Brain Res* 2010, **1366**:172-188.

16. Lee SY, Patton SM, Henderson RJ, Connor JR: **Consequences of expressing mutants of the hemochromatosis gene (HFE) into a human neuronal cell line lacking endogenous HFE**. *FASEB J* 2007, **21**(2):564-576.

17. Meneses-Lorente G, Guest PC, Lawrence J, Muniappa N, Knowles MR, Skynner HA, Salim K, Cristea I, Mortishire-Smith R, Gaskell SJ, [Watt A](http://www.ncbi.nlm.nih.gov/pubmed?term="Watt A"%5BAuthor%5D):**A proteomic investigation of drug-induced steatosis in rat liver**. *Chem Res Toxicol* 2004, **17**(5):605-612.

18. Schulte J, Struck J, Köhrle J, Müller B: **Circulating levels of peroxiredoxin 4 as a novel biomarker of oxidative stress in patients with sepsis**. *Shock* 2011, **35**(5):460-465.

19. Wang X, Zhang S, Sun C, Yuan ZG, Wu X, Wang D, Ding Z, Hu R: **Proteomic profiles of mouse neuro n2a cells infected with variant virulence of rabies viruses**. *J Microbiol Biotechnol* 2011, **21**(4):366-373.

20. Yu S, Mu Y, Ao J, Chen X: **Peroxiredoxin IV regulates pro-inflammatory responses in large yellow croaker (Pseudosciaena crocea) and protects against bacterial challenge**. *J Proteome Res* 2010, **9**(3):1424-1436.

21. Jamaluddin M, Wiktorowicz JE, Soman KV, Boldogh I, Forbus JD, Spratt H, Garofalo RP, Brasier AR: **Role of peroxiredoxin 1 and peroxiredoxin 4 in protection of respiratory syncytial virus-induced cysteinyl oxidation of nuclear cytoskeletal proteins**. *J Virol* 2010, **84**(18):9533-9545.

22. Knight M, Raghavan N, Goodall C, Cousin C, Ittiprasert W, Sayed A, Miller A, Williams DL, Bayne CJ: **Biomphalaria glabrata peroxiredoxin: effect of schistosoma mansoni infection on differential gene regulation**. *Mol Biochem Parasitol* 2009, **167**(1):20-31.

23. Mota S, Mendes M, Freitas N, Penque D, Coelho AV, Cunha C: **Proteome analysis of a human liver carcinoma cell line stably expressing hepatitis delta virus ribonucleoproteins**. *J Proteomics* 2009, **72**(4):616-627.

24. Jin D-Y, Chae HZ, Rhee SG, Jeang K-T: **Regulatory role for a novel human thioredoxin peroxidase in NF-kB activation**. *J Biol Chem* 1997, **272**(49):30952–30961.

25. Dreja T, Jovanovic Z, Rasche A, Kluge R, Herwig R, Tung YC, Joost HG, Yeo GS, Al-Hasani H: **Diet-induced gene expression of isolated pancreatic islets from a polygenic mouse model of the metabolic syndrome**. *Diabetologia* 2010, **53**(2):309-320.

26. Jiang YL, Ning Y, Ma XL, Liu YY, Wang Y, Zhang Z, Shan CX, Xu YD, Yin LM, Yang YQ: **Alteration of the proteome profile of the pancreas in diabetic rats induced by streptozotocin**. *Int J Mol Med* 2011, **28**(2):153-160.

27. Xie X, Li S, Liu S, Lu Y, Shen P, Ji J: **Proteomic analysis of mouse islets after multiple low-dose streptozotocin injection**. *Biochim Biophys Acta* 2008, **1784**(2):276-284.

28. Huo R, He Y, Zhao C, Guo X-J, Lin M, Sha J-H: **Identification of human spermatogenesis-related proteins by comparative proteomic analysis: a preliminary study**. *Fertil Steril* 2008, Oct;90(4):1109-18.

29. Matsuki S, Sasagawa I, Iuchi Y, Fujii J: **Impaired expression of peroxiredoxin 4 in damaged testes by artificial cryptorchidism**. *Redox Rep* 2002, **7**(5):276-278.

30. Godoy JR, Oesteritz S, Hanschmann EM, Ockenga W, Ackermann W, Lillig CH: **Segment-specific overexpression of redoxins after renal ischemia and reperfusion: protective roles of glutaredoxin 2, peroxiredoxin 3, and peroxiredoxin 6**. *Free radical biology & medicine* 2011, **51**(2):552-561.

31. Nickel CH, Ruedinger J, Misch F, Blume K, Maile S, Schulte J, Köhrle J, Hartmann O, Giersdorf S, Bingisser R: **Copeptin and peroxiredoxin-4 independently predict mortality in patients with nonspecific complaints presenting to the emergency department**. *Acad Emerg Med* 2011, **18**(8):851-859.
